# Inter-leg systolic blood pressure difference has been associated with all-cause and cardiovascular mortality: analysis of NHANES 1999–2004

**DOI:** 10.1186/s12889-024-18508-8

**Published:** 2024-04-17

**Authors:** Geng Shen, Zhihao Liu, Leyi Wang, Jianping Li

**Affiliations:** Division of Cardiology, Peking University First Hospital, No. 8 Xishiku St, Xicheng District, 100034 Beijing, China

**Keywords:** Inter-leg systolic blood pressure difference, All-cause mortality, Cardiovascular mortality, Centers for Disase Control and Prevention, National Health and Nutrition Examination Survey

## Abstract

**Background:**

Inter-leg systolic blood pressure difference (ILSBPD) has emerged as a novel cardiovascular risk factor. This study aims to investigate the predictive value of ILSBPD on all-cause and cardiovascular mortality in general population.

**Methods:**

We combined three cycles (1999–2004) of the National Health and Nutrition Examination Survey (NHANES) data. Levels of ILSBPD were calculated and divided into four groups based on three cut-off values of 5, 10 and 15mmHg. Time-to-event curves were estimated with the use of the Kaplan-Meier method, and two multivariable Cox proportional hazards regression models were conducted to assess the hazard ratios (HRs) and 95% confidence intervals (CIs) of all-cause and cardiovascular mortality associated with ILSBPD.

**Results:**

A total of 6 842 subjects were included, with the mean (SD) age of 59.5 (12.8) years. By December 31, 2019, 2 544 and 648 participants were identified all-cause and cardiovascular mortality respectively during a median follow-up of 16.6 years. Time-to-event analyses suggested that higher ILSBPD was associated with increased all-cause and cardiovascular mortality (logrank, *p* < 0.001). Every 5mmHg increment of ILSBPD brings about 5% and 7% increased risk of all-cause and cardiovascular mortality, and individuals with an ILSBPD ≥ 15mmHg were significantly associated with higher incidence of all-cause mortality (HR 1.43, 95%CI 1.18–1.52, *p* < 0.001) and cardiovascular mortality (HR 1.73, 95%CI 1.36–2.20, *p* < 0.001) when multiple confounding factors were adjusted. Subgroup and sensitivity analysis confirmed the relationship.

**Conclusions:**

Our findings suggest that the increment of ILSBPD was significantly associated with higher risk of all-cause and cardiovascular mortality in general population.

**Supplementary Information:**

The online version contains supplementary material available at 10.1186/s12889-024-18508-8.

## Introduction

Cardiovascular disease (CVD) is the most common non-communicable disease worldwide that causes substantial economic and social burdens [1, 2]. However, traditional risk assessment fell short in accurately identifying all CVD risk factors [3–7], and researchers are currently gaining more understanding toward residual cardiovascular risks to further achieve early identification and precise intervention.

Inter-leg systolic blood pressure difference (ILSBPD), one of the parameters derived from four-limb blood pressure measurement that stands for the difference in systolic blood pressure between the lower extremities, has emerged with impressively predictive value of impaired vascular function and was linked to cardiovascular risk factors, target organ damage, stroke etc [8–17].. However, only a small number of studies explored the association between ILSBPD and mortality outcomes, with limited sample sizes and the observed population restricted to the elderly [18], or patients with hemodialysis as well as those with acute myocardial infarction [19–21].

Therefore, there remains a scarcity of large-scale observational studies examining the role of ILSBPD in predicting clinical outcomes, especially its application potential on the general population warrants further investigation. To address the gaps of previous research, this study retrospectively examined the association of ILSBPD with risk of all-cause and cardiovascular mortality in a representative database of general population.

## Methods

Data utilized in this study are openly available by the National Center for Health Statistics at https://www.cdc.gov/nchs/nhanes/index.htm.

### Study population

The National Health and Nutrition Examination Survey (NHANES) is an ongoing survey to assess the health and nutritional status of general population in the United States, with cross-sectional studies conducted continuously in 2-year cycles by the National Center for Health Statistics [22]. Demographic information, medical history, and health-related behaviors were collected through standardized questionnaires at study recruitment, while physical examinations, laboratory tests and post-examination follow-ups were as well performed by highly-trained professionals [23]. All protocols were approved by the National Center for Health Statistics Ethics Review Board, and informed consent was obtained from all study participants. This study followed the Strengthening the Reporting of Observational Studies in Epidemiology (STROBE) reporting guideline for cohort studies [24].

The present investigation included participants enrolled in three two-year cycles of NHANES data, spanning from 1999 to 2004, when the lower extremity disease (LED) component was conducted and thus data concerning blood pressure of lower extremities was available. The exclusion criteria were: (a) lack of complete data on blood pressure of the lower extremities (*n* = 24 272); (b) lack of follow-up data (*n* = 11); (c) cause of death unknown (*n* = 1). A total of 6 842 participants were included in the study (details see Supplementary Fig. 1).

### Measurement of ILSBPD

Blood pressure was assessed in the supine position of all participants using a vascular testing device (Parks Mini-Lab IV, Model 3100; Aloha, OR) on the right arm and both ankles. Two measurements were taken for subjects aged 40–59 years, while only one measurement was taken for subjects who were aged 60 years and older [25]. The term ILSBPD refers to the absolute value of the systolic blood pressure (SBP) difference between the lower extremities.

### Outcome follow-up

The data from NHANES has been linked to the death certificate records of the National Death Index (NDI) by The National Center for Health Statistics (NCHS). Individuals aged 18 years or older with sufficient identifying information were included in the study for mortality follow-up, which began at the date of survey participation and ended at the date of death or study censoring (December 31, 2019). The underlying causes of death were determined using the International Classification of Diseases, Tenth Revision (ICD-10). The outcome of this study was all-cause mortality and cardiovascular mortality.

### Assessment of covariates

Demographic and lifestyle variables were obtained using a standardized participant questionnaire in the NHANES database. This included a household interview, two 24-hour recall interviews, and a medical evaluation of each participant’s key characteristics, including age, gender (male or female), ethnicity (Hispanic, non-Hispanic White, non-Hispanic Black, and others, which included other Hispanic, other non-Hispanic race, and non-Hispanic multiracial), smoking status (non-smoker, less than 100 cigarettes in the lifetime; smoker, over 100 cigarettes in the lifetime), drinking status (non-heavy drinker, less than 5 drinks per day; heavy drinker, more than 5 drinks per day) etc. SBP was measured on right arm and body mass index (BMI) was calculated as body weight in kilogram divided by squared height (kg/m2). Blood test of total cholesterol (TCHO), high-density lipoprotein cholesterol (HDL-c), as well as glycated hemoglobin (HbA1c) were obtained through laboratory analysis.

### Statistical analysis

The data were processed in accordance with NHANES analytical guidelines, and analyses were performed using EmpowerStats (http://www.empowerstats.com) and the statistical package R (4.2.0 version). In the baseline demographic characteristics, continuous variables that followed a normal distribution were reported as means ± standard deviations (SD) and compared between two groups using the Student’s t-test. Categorical variables were presented as frequencies and percentages, and differences between groups were evaluated by means of the Chi-square test.

Levels of ILSBPD was divided into four groups based on the three cut-off values of 5, 10 and 15mmHg. Time-to-event curves were estimated with the use of the Kaplan-Meier method, and the multivariable Cox proportional hazards regression model was conducted to assess the hazard ratios (HRs) and 95% confidence intervals (CIs) for the associations between ILSBPD and risks of all-cause and cardiovascular mortality. Schoenfeld residuals were used to test the proportional hazards assumption, and no violation was observed. Crude analysis was performed (model 1) and two multivariable models were constructed. Model 2 was adjusted for age (continuous, years), gender (male or female), and race and ethnicity (Mexican American, non-Hispanic Black, non-Hispanic White, or others). In model 3, we further adjusted for BMI (continuous), systolic blood pressure on right arm (continuous), smoking status (non-smoker or smoker), drinking status (non-heavy drinker or heavy drinker), TCHO (continuous), HDL-c (continuous), and HbA1c (continuous).

In subgroup analyses, we exploratorily investigated the modifying effect of the association between ILSBPD with all-cause and cardiovascular mortality. Interactions were examined by including interaction terms in the regression models. We further stratified the analyses by age (< 60, 60 -< 70, or ≥ 70 years), gender (male or female), race and ethnicity (Mexican American, non-Hispanic Black, non-Hispanic White, or others), BMI (< 28 or ≥ 28), smoking status (non-smoker or smoker), alcohol consumption (non-heavy drinker or heavy drinker), brachial SBP (< 140 or ≥ 140 mmHg), TCHO (< 5.2 or ≥ 5.2 mmol/L), HDL-c (< 1.04 or ≥ 1.04 mmol/L), and HbA1c (< 7% or ≥ 7%). The *p* values for the production terms between ILSBPD and the stratified factors were used to estimate the significance of interactions.

Further, sensitivity analyses were performed to evaluate the robustness of the results. Firstly, participants who died within 2 years of follow-up were excluded to minimize the possibility of reverse-causality bias. Secondly, as peripheral artery disease (PAD) might influence ILSBPD, we further adjusted PAD (defined as ankle-brachial index [ABI] < 0.9) in the new model. Moreover, to assess the potential relationship between ILSBPD and ABI, we further performed a second sensitivity analysis with ABI adjusted as a continuous variable on the basis of a fully adjusted model. In this analysis, we utilized the blood pressure measured at the first time for each patient to eliminate the potential bias induced by the times of blood pressure measurement (one time for individuals ≥ 60 years and two times for 40–59 years). A two-sided *P* value of < 0.05 was considered significant.

## Results

### Characteristics of study participants

A total of 31 116 subjects were surveyed during the continuous NHANES cycles between 1999 and 2004. As shown in Supplementary Fig. 1, an overall of 6 842 participants (3 514 male [51.4%]) aged 40 years or more who had measured systolic blood pressure of lower extremities were included in the analysis. Baseline demographic and clinical characteristics of the study participants were presented in Table 1. The mean (SD) age of the population was 59.5 (12.8) years. At the census date (December 31, 2019), respectively 2 544 (37.2%) and 1 648 (9.5%) participants were recorded to have gone through all-cause and cardiovascular mortality.

**Table 1:** Baseline characteristics of included participants from NHANES 1999 through 2004

Characteristics	Participants *N* = 6842	ILSBPD range (mmHg)				*p*-value
		Group 1 (< 5) *n* = 2996	Group 2 (5 -< 10) *n* = 1928	Group 3 (10 -< 15) *n* = 935	Group 4 (≥ 15) *n* = 983	
Age (years)	59.5 ± 12.8	57.1 ± 12.1	59.5 ± 12.7	61.0 ± 12.8	65.6 ± 12.9	< 0.001
Gender						0.341
Male	3514 (51.4%)	1511 (50.4%)	985 (51.1%)	496 (53.0%)	522 (53.1%)	
Female	3328 (48.6%)	1485 (49.6%)	943 (48.9%)	439 (47.0%)	461 (46.9%)	
Race/ethnicity						0.17
Mexican American	1451 (21.2%)	622 (20.8%)	441 (22.9%)	175 (18.7%)	213 (21.7%)	
Other Hispanic	467 (6.8%)	203 (6.8%)	137 (7.1%)	74 (7.9%)	53 (5.4%)	
non-Hispanic white	3713 (54.3%)	1626 (54.3%)	1030 (53.4%)	517 (55.3%)	540 (54.9%)	
non-Hispanic black	1211 (17.7%)	545 (18.2%)	320 (16.6%)	169 (18.1%)	177 (18.0%)	
BMI (kg/m2)	28.4 ± 5.6	28.3 ± 5.7	28.4 ± 5.5	28.4 ± 5.5	28.6 ± 5.7	0.631
SBP average (mmHg)	130.7 ± 20.7	128.3 ± 20.2	129.9 ± 19.6	132.8 ± 20.0	138.1 ± 23.3	< 0.001
TCHO (mmol/L)	5.4 ± 1.1	5.4 ± 1.0	5.4 ± 1.1	5.4 ± 1.0	5.4 ± 1.1	0.455
HDL-c (mmol/L)	1.4 ± 0.4	1.4 ± 0.4	1.4 ± 0.4	1.4 ± 0.4	1.4 ± 0.4	0.84
HbA1c (%)	5.8 ± 1.1	5.7 ± 1.1	5.8 ± 1.2	5.8 ± 1.1	5.9 ± 1.2	< 0.001
Smoked at least 100 cigarettes in life						0.113
Yes	3705 (54.2%)	1597 (53.3%)	1032 (53.6%)	512 (54.9%)	564 (57.6%)	
No	3128 (45.8%)	1397 (46.7%)	895 (46.4%)	421 (45.1%)	415 (42.4%)	
Ever have 5 or more drinks every day						< 0.001
Yes	1068 (18.6%)	427 (16.9%)	289 (17.9%)	166 (21.1%)	186 (22.7%)	
No	4677 (81.4%)	2100 (83.1%)	1322 (82.1%)	621 (78.9%)	634 (77.3%)	
All-cause mortality						< 0.001
No	4298 (62.8%)	2091 (69.8%)	1219 (63.2%)	562 (60.1%)	426 (43.3%)	
Yes	2544 (37.2%)	905 (30.2%)	709 (36.8%)	373 (39.9%)	557 (56.7%)	
Cardiovascular mortality						< 0.001
No	6194 (90.5%)	2787 (93.0%)	1757 (91.1%)	848 (90.7%)	802 (81.6%)	
Yes	648 (9.5%)	209 (7.0%)	171 (8.9%)	87 (9.3%)	181 (18.4%)	

Data is presented with mean (SD) or number of participants (percentage)

*p* < 0.05 indicate significant difference between or across groups

Abbreviations: NHANES: National Health and Nutrition Examination Survey; ILSBPD: inter-leg systolic blood pressure difference; BMI: body mass index; SBP: systolic blood pressure; TCHO: total cholesterol; HDL-c: high density lipoprotein cholesterol; HbA1c: glycated hemoglobin

Participants with an ILSBPD ≥ 15 mmHg tended to be older, with higher SBP on right arm and higher levels of HbA1c, as well as higher rate of being heavy drinkers, and they were consequently more likely to have gone through all-cause and cardiovascular deaths. No significant difference was observed between the four groups categorized according to ILSBPD regarding gender, ethnicity, BMI, smoking status, TCHO and HDL-c levels.

### Associations between ILSBPD and all-cause mortality

**Fig. 1:**
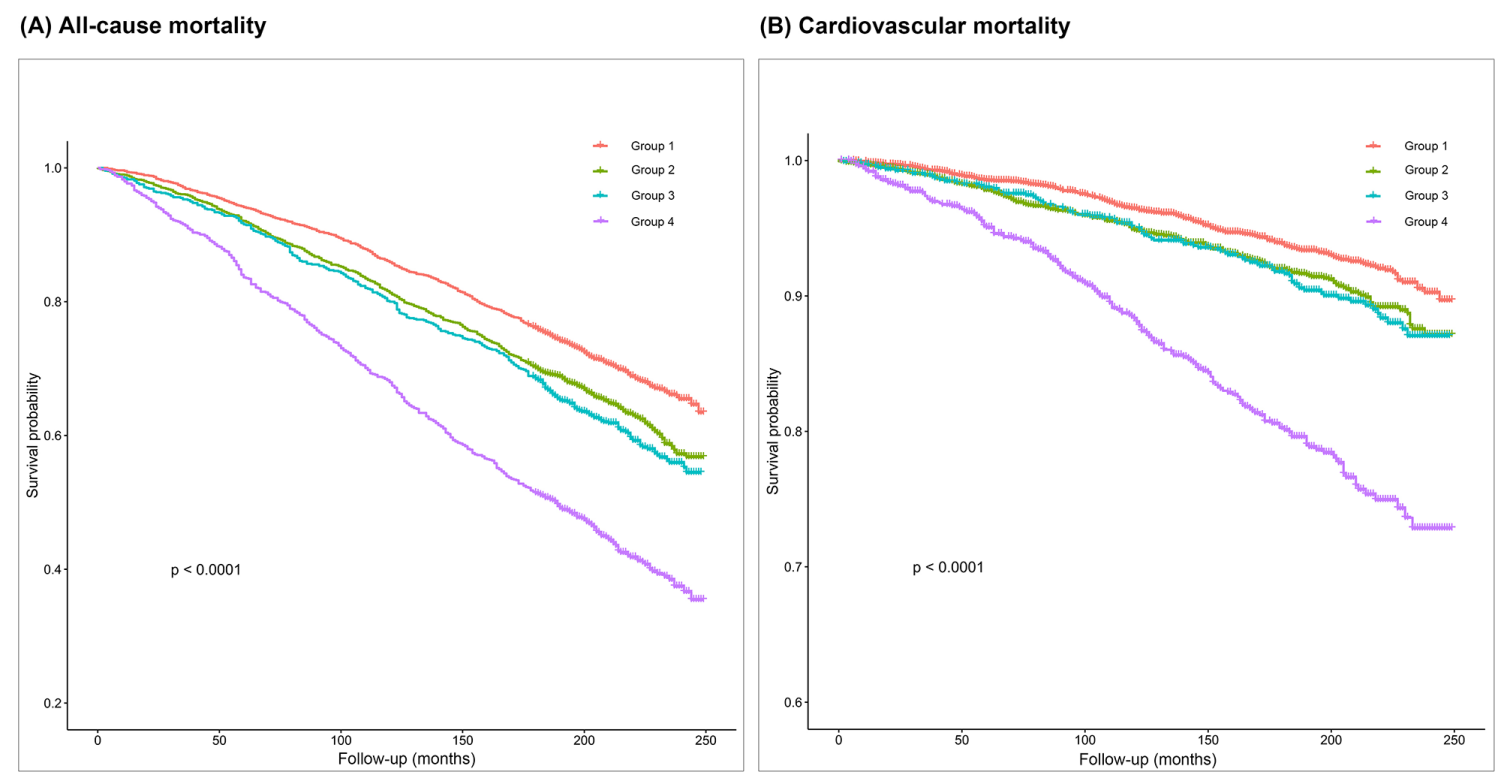
Kaplan-Meier estimates for outcome events. Panels present the Kaplan-Meier curves for (**A**) all-cause mortality and (**B**) cardiovascular mortality in 6 842 participants divided into four groups according to ILSBPD

During a median follow-up of 16.6 years, 2 544 (37.2%) participants died among 6 842 individuals. Time-to-event analyses are shown in Fig. 1(A). Kaplan-Meier curve suggested that higher ILSBPD was associated with increased all-cause mortality (logrank, *p* < 0.0001).

Table 2 shows the results of Cox regression analyses of the association between ILSBPD and all-cause mortality. When considered as a continuous variable, every 5mmHg increment of ILSBPD brings about 16%, 6% and 5% increased risk of all-cause mortality in crude model (model 1), model 2 which adjusted for demographic factors, and model 3 which further adjusted for multiple confounding factors, respectively (*p* < 0.001 for each model). While categorizing ILSBPD into four groups with the three cut-off values of 5, 10 and 15mmHg, all three groups (ILSBPD between 5 -< 10mmHg, 10 -< 15mmHg and ≥ 15mmHg) manifested significant association with all-cause mortality compared with individuals with ILSBPD < 5mmHg in unadjusted model (Table 2, model 1). Nevertheless, only individuals with ILSBPD ≥ 15mmHg were significantly associated with increased incidence of going through all-cause mortality when demographic factors (model 2, HR 1.38, 95%CI 1.24–1.54, *p* < 0.001) and multiple confounding factors were adjusted (model 3, HR 1.34, 95%CI 1.18–1.52, *p* < 0.001), but for all models, it remained consistent that the risk manifested a significant upward trend across groups as ILSBPD increased (p for trend < 0.001).

**Table 2:** Cox regression analyses of the association between ILSBPD and all-cause and cardiovascular mortality

Model	Hazard ratio (95% confidence interval), *p* value					
	Inter-leg systolic blood pressure, mmHg					
	Continuous (per 5mmHg)	Groups				
		Group 1 (< 5)	Group 2 (5 -< 10)	Group 3 (10 -< 15)	Group 4 (≥ 15)	*p* for trend
**All-cause mortality**						
Deaths, No. /total, No. (%)	2544/6842 (37.2)	905/2996 (30.2)	709/1928 (36.8)	373/935 (39.9)	557/983 (56.7)	
Model 1 *	1.16 (1.15, 1.18), *p* < 0.001	ref	1.27 (1.16, 1.41), *p* < 0.001	2.39 (2.15, 2.66), *p* < 0.001	3.37 (2.76, 4.11), *p* < 0.001	*p* < 0.001
Model 2 †	1.06 (1.04, 1.08), *p* < 0.001	ref	1.09 (0.99, 1.21), *p* = 0.0791	1.04 (0.92, 1.18), *p* = 0.4904	1.38 (1.24, 1.54), *p* < 0.001	*p* < 0.001
Model 3 ‡	1.05 (1.03, 1.07), *p* < 0.001	ref	1.05 (0.94, 1.18), *p* = 0.3674	1.03 (0.90, 1.19), *p* = 0.6749	1.34 (1.18, 1.52), *p* < 0.001	*p* < 0.001
**Cardiovascular mortality**						
Deaths, No. /total, No.	648/6842 (9.5%)	209/2996 (7.0%)	171/1928 (8.9%)	87/935 (9.3%)	181/983 (18.4%)	
Model 1 *	1.20 (1.17, 1.24), *p* < 0.001	ref	1.33 (1.09, 1.63), *p* = 0.006	1.41 (1.10, 1.81), *p* = 0.007	3.37 (2.76, 4.11), *p* < 0.001	*p* < 0.001
Model 2 †	1.09 (1.06, 1.12), *p* < 0.001	ref	1.13 (0.92, 1.38), *p* = 0.246	1.02 (0.79, 1.31), *p* = 0.890	1.83 (1.49, 2.24), *p* < 0.001	*p* < 0.001
Model 3 ‡	1.07 (1.04, 1.11), *p* < 0.001	ref	1.15 (0.90, 1.45), *p* = 0.259	1.01 (0.76, 1.36), *p* = 0.923	1.73 (1.36, 2.20), *p* < 0.001	*p* < 0.001

Data is presented with mean (SD) or number of participants (percentage)

*p* < 0.05 indicate significant difference between or across groups

* Crude model

† Adjusted for age, gender, and race/ ethnicity

‡ Further adjusted for BMI, smoking status, systolic blood pressure on right arm, smoking status, drinking status, TCHO, HDL-c and HbA1c

Abbreviations: ILSBPD: inter-leg systolic blood pressure difference; NHANES: National Health and Nutrition Examination Survey; BMI: body mass index; SBP: systolic blood pressure; TCHO: total cholesterol; HDL-c: high density lipoprotein cholesterol; HbA1c: glycated hemoglobin

### Associations between ILSBPD and cardiovascular mortality

648 (9.5%) in 6 842 individuals died of cardiovascular causes during follow-up, and Kaplan-Meier curve revealed that higher ILSBPD was as well associated with increased cardiovascular mortality (logrank, *p* < 0.0001, Fig. 1[B]).

Cox regression analyses were performed and the results indicated a higher incidence of cardiovascular mortality to have been associated with higher ILSBPD, as manifested in Table 2. Every 5mmHg increment of ILSBPD brings out 20%, 9% and 7% increased risk of cardiovascular mortality in model 1, 2 and 3, respectively, when considered as a continuous variable (*p* < 0.001 for every model). With regard to categorized ILSBPD, individuals with an ILSBPD ≥ 15 mmHg were associated with an increased incidence of cardiovascular mortality in crude model (HR 3.37, 95%CI 2.76–4.11, *p* < 0.001), as well in model 2 (HR 1.83, 95%CI 1.49–2.24, *p* < 0.001) and model 3 (HR 1.73, 95%CI 1.36–2.20, *p* < 0.001), and the risk significantly increased across groups (p for trend < 0.001) in all models.

### Subgroup analysis

**Fig. 2:**
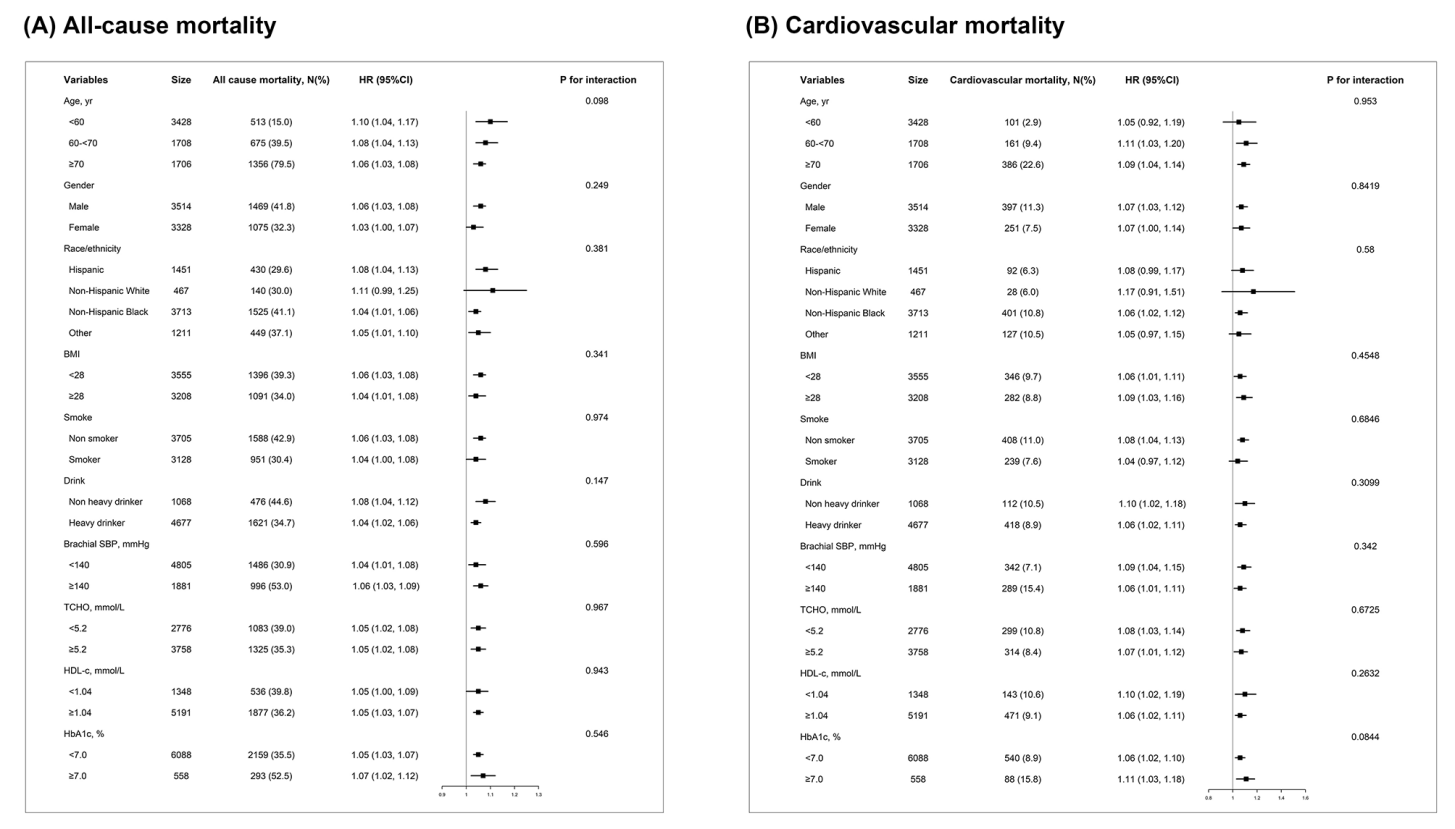
Subgroup analysis on the association between ILSBPD and all-cause and cardiovascular mortality Subgroup analysis according to multiple clinical variables to identify any modification effect on the association between ILSBPD and either all-cause mortality or cardiovascular mortality. No interaction was observed. HRs have been fully adjusted for age, gender, race/ethnicity, BMI, systolic blood pressure on right arm, smoking status, drinking status, TCHO, HDL-c and HbA1c. *p* < 0.05 indicate significant difference Abbreviations: ILSBPD: inter-leg blood pressure difference; HR: hazard ratio; BMI: body mass index; SBP: systolic blood pressure; TCHO: total cholesterol; HDL-c: high density lipoprotein cholesterol; HbA1c: glycated hemoglobin

Subgroup analysis did not identify any modification of the effect of ILSBPD on either all-cause mortality or cardiovascular mortality according to multiple clinical variables, including traditional cardiovascular risk factors (Fig. 2), indicating that the associations between ILSBPD with all-cause and cardiovascular mortality were generally consistent across subgroups.

### Sensitivity analysis

The results remained consistent as we excluded participants who died within 2 years of follow-up meanwhile adjusted for PAD (Supplementary Table 1). Moreover, when we adopted the first-time measured blood pressure to calculate ILSBPD and performed adjustment according to ABI, we still gained a significant association between ILSBPD and cardiovascular mortality, nevertheless, the result was no longer significant regarding all-cause mortality (Supplementary Table 2).

## Discussion

In this large-scale population-based cohort study exploring the association of ILSBPD with all-cause and cardiovascular death, we discovered that ILSBPD was positively associated with all cause and cardiovascular death independent of age, gender, ethics and traditional cardiovascular risk factors.

Previous studies demonstrated relationships between ILSBPD and cardiovascular risk factors as well as target organ damage, with observed association with PAD, left ventricular mass index, brachial-ankle pulse wave velocity (baPWV) and kidney function revealed by eGFR [8, 9, 13–26]. However, large-scale investigations regarding clinical outcomes are lacking. A study involving 210 hemodialysis patients with a mean follow-up of 4.4 ± 1.5 years discovered that ILSBPD ≥ 15mmHg was an independent predictor for overall mortality (HR 2.91, 95% CI 1.28–6.64, *p* = 0.01) and cardiovascular mortality (HR 3.15, 95% CI 1.05–9.44, *p* = 0.04)^31^. Similar results were obtained in incident dialysis patients [19]. In an elderly Chinese population, ILSBPD was proved to be an independent risk factor of total mortality and cardiovascular mortality [27]. For patients undergoing percutaneous intervention, increased ILSBPD was independently associated with major adverse cardiovascular events (MACE; per 5mmHg; HR 1.07; 95% CI, 1.01–1.14) [28]. Another cross-sectional study included 1 485 participants demonstrated that the addition of ILSBPD ≥ 10mmHg to the traditional risk factors improved the prediction efficacy of stroke [29].

While prior research had hinted at the potential role of ILSBPD as a risk factor for cardiovascular diseases and adverse clinical outcomes, many of these studies primarily focused on single ethnic populations or with specific participant restrictions (e.g., the elderly, hemodialysis patients, patients with atherosclerotic cardiovascular disease [ASCVD] etc.), and the predictive value of ILSBPD in general population remained unexplored. Our study sought to contribute to the existing body of evidence by examining a general population encompassing various genetic backgrounds. Furthermore, the limited sample sizes and short follow-up durations in previous investigations may have compromised the robustness of their conclusions. In contrast, the current study utilized NHANES database, featuring substantial observational data and long follow-up periods, addressing the shortcomings of earlier research.

Our findings strongly suggest a positive correlation between ILSBPD and both all-cause and cardiovascular mortality, with an observed increased risk of 5% and 7%, respectively, for each 5mmHg increment in ILSBPD. Furthermore, individuals with an ILSBPD ≥ 15mmHg exhibited a notably higher incidence of all-cause and cardiovascular mortality, with respectively 34% and 73% increased risk. These trends were also clearly illustrated in Kaplan-Meier plots. A prospective cohort study conducted by Sheng et al. reported higher HRs for ILSBPD concerning the same clinical outcomes. In their study, an increase in ILSBPD of 1 standard deviation (6.4mmHg) corresponded to a 15% increased risk of all-cause mortality and a 20% increased risk of cardiovascular mortality [18]. Several factors may account for these variations, including differences in the ethnic backgrounds of study participants, the covariates adjusted for in Cox regression models, and the potential influence of confounding fundamental diseases, given that Sheng et al.‘s study primarily involved elderly population.

A universally accepted cutoff point for ILSBPD has not yet been established. In a community-based study conducted by Zhang et al., a suggested upper limit of 16.7mmHg was proposed as the normal threshold for ILSDBP [30]. Furthermore, many related studies have opted for cutoff values of 10 or 15mmHg for categorization [10, 11, 13, 18, 29, 31, 32]. On the basis of these prior investigations, our study employed cutoff points of 5, 10, and 15mmHg, taking both their clinical simplicity and previous research findings into consideration. The results revealed that neither the group with ILSBPD between 5 -< 10mmHg nor the group with ILSBPD between 10 -< 15mmHg exhibited a significant increase in the incidence of all-cause mortality or cardiovascular mortality when compared to the group with ILSBPD < 5mmHg. However, noteworthy increased risks were observed in individuals with ILSBPD ≥ 15mmHg. Therefore, these findings suggest that an ILSBPD value of 15mmHg could potentially serve as an effective cutoff point for predicting all-cause and cardiovascular mortality within general population.

Subgroup analysis provided additional robustness to the observed relationship between ILSBPD and clinical outcomes, proving the results solid regardless of age, gender, race or other cardiovascular risk factors.

Furthermore, considering the significant relevance of ILSBPD to PAD, as well that PAD has been recognized as an independent risk factor for both all-cause and cardiovascular mortality, it became crucial to assess whether the results could remain consistent when putting asides patients with PAD. Sheng et al. previously reported that the exclusion of patients with an ABI less than 0.9 did not alter the predictive value of ILSBPD on mortality [18]. In line with this, patients suspected of PAD (defined as ABI < 0.9) were as well excluded in our sensitivity analysis, and the result turned out that the association between ILSBPD and mortality could be well independent of PAD. Nevertheless, we further made a more in-depth exploration by adjusting ABI as a continuous variable. The result remained significant for cardiovascular mortality but lost statistical significance in the context of all-cause mortality, suggesting that a potential correlation between ILSPBPD and PAD could not be completely overlooked. A mediation analysis could be conducted to further quantitatively assess the potential mediation effect of PAD between ILSBPD and all-cause and cardiovascular mortality.

Compared with inter-arm systolic blood pressure difference (IASBPD), the difference in systolic blood pressure between the upper limbs, another novel indicator associated with increased risks of vascular diseases and mortality beyond traditional cardiovascular risk factors [33–37], ILSBPD has been demonstrated to be of higher predictive value on PAD and MACE [13, 28]. The difference in lengths between arterial pathways in upper and lower limbs may account for the disparity between IASBPD and ILSBPD. Longer arteries are more likely to be exposed to pathologic vessel conditions (e.g., atherosclerosis), thus better reveal the burden of vascular diseases and could serve as a more precise parameter in visualizing the severity of vascular lesions with differences in blood pressure.

ILSBPD emerges as a valuable clinical parameter with predictive capabilities for both all-cause and cardiovascular mortality in general population, complementing traditional cardiovascular risk factors while assessing residual risks for mortality.

Furthermore, as the measurement of ILSBPD has been easily accessible, it could be especially valuable in regions where advanced medical techniques or skilled technicians may not be readily available. Moreover, in patients going through dialysis or upper limb bone fracture etc., ILSBPD could be considered as a proper replacement of IASBPD with high predictive value on clinical outcomes.

This study has some notable strengths. The present study is the first investigation on the association of ILSBPD and all-cause and cardiovascular mortality in general population based on the multiethnic NHANES database with consideration of a multitude of potential confounding factors.

This study as well has a few potential limitations. First, the data of lower extremity blood pressure was only available in the survey data collected from 1999 to 2004, and only adults over 40 years old were included. Second, the blood pressure of lower extremities for subjects who were aged 60 years and older were based on only single measurement, which may not accurately reflect the long-term blood pressure status. Third, covariates collected at baseline may change over time, meanwhile residual or unknown confounding factors cannot be entirely excluded, thus leading to a potential influence on the association of ILSBPD with all-cause and cardiovascular mortality. Forth, owing to the nature of the observational study design, our findings cannot be used for inference of causality. Fifth, as causes of death were determined by the NCHS using ICD-10, misclassification or undiagnosed cardiovascular cause of death might have been inevitable.

## Conclusions

In conclusion, our study reveals that within general population, ILSBPD emerges as an independent risk factor for both all-cause and cardiovascular mortality.

### Electronic supplementary material

Below is the link to the electronic supplementary material.

Supplementary Material 1

## Data Availability

Data utilized in this study are openly available by the National Center for Health Statistics at https://www.cdc.gov/nchs/nhanes/index.htm.

## Abbreviations

ABI: Ankle-brachial index
BMI: Body mass index
HbA1c: Glycated hemoglobin
HDL-c: High density lipoprotein cholesterol
IASBPD: Inter-arm systolic blood pressure difference
ILSBPD: Inter-leg blood pressure difference
PAD: Peripheral artery disease
SBP: Systolic blood pressure
TCHO: Total cholesterol
