# Supplementary material for: Inter-leg systolic blood pressure difference has been associated with all-cause and cardiovascular mortality: analysis of NHANES 1999–2004
Source: BMC Public Health. 2024 Apr 17;24:1071. doi: 10.1186/s12889-024-18508-8 (PMC11025152; doi:10.1186/s12889-024-18508-8)
Supplement: Supplementary file 1 — Supplementary Material 1 [file 12889_2024_18508_MOESM1_ESM.docx]

**Supplementary Information**

**Supplementary figure 1.** Flow chart illustrating the inclusion and exclusion of study participants.

**
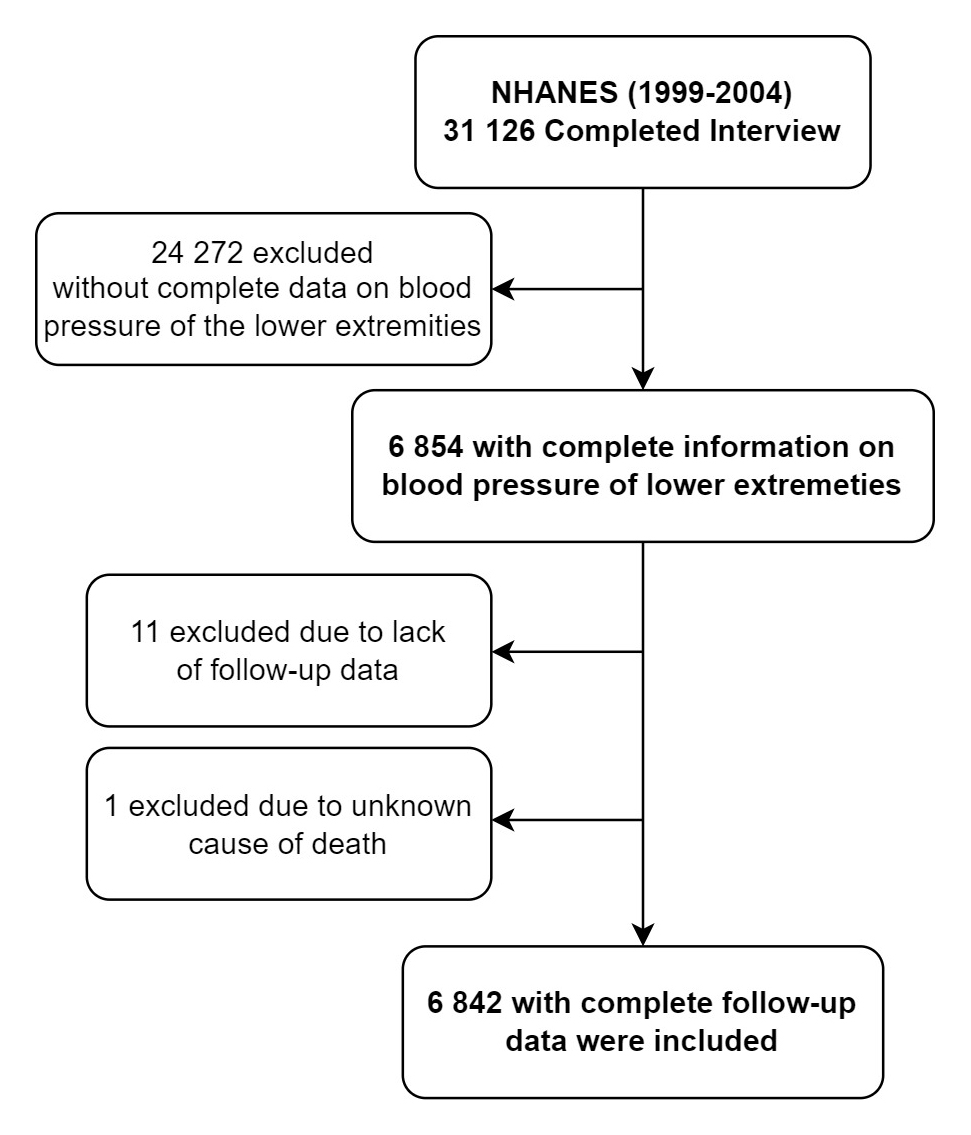
**

**Supplementary table 1.** Sensitivity analyses of the associations (hazard ratios, 95% confidence intervals) between ILSBPD and the risk of all-cause and cardiovascular mortality in general population (NHANES 1999-2004), by excluding participants who died within 2 years of follow-up and adjusted for PAD.

| Model | Hazard ratio (95% CI), p value | | | | |
| --- | --- | --- | --- | --- | --- |
|  | Inter-leg systolic blood pressure, mmHg | | | | |
|  | Group 1  (<5) | Group 2  (5-<10) | Group 3  (10-<15) | Group 4  (≥15) | P for trend |
| **All-cause mortality** | | | | | |
| Deaths, No. /total, No. (%) | 820/2886 (28.4%) | 614/1816 (33.8%) | 299/834 (35.9%) | 304/660 (46.1%) |  |
| Model 1 ^a^ | ref | 1.23 (1.10, 1.36), p<0.001 | 1.29 (1.13, 1.47), p<0.001 | 1.86 (1.63, 2.12), p<0.001 | <0.001 |
| Model 2^b^ | ref | 1.07 (0.96, 1.18), p=0.230 | 0.95 (0.83, 1.09), p=0.456 | 1.18 (1.04, 1.35), p=0.013 | 0100 |
| Model 3^c^ | ref | 1.03 (0.91, 1.16), p=0.617 | 0.94 (0.81, 1.10), p=0.447 | 1.20 (1.03, 1.40), p=0.019 | 0.129 |
| **Cardiovascular mortality** | | | | | |
| Deaths, No. /total, No. (%) | 187/2886  (6.5%) | 143/1816 (7.9%) | 68/834  (8.2%) | 94/660 (14.2%) |  |
| Model 1 ^a^ | ref | 1.25 (1.01, 1.56), p=0.043 | 1.29 (0.97, 1.70), p=0.077 | 2.52 (1.97, 3.23), p<0.0001 | <0.001 |
| Model 2^b^ | ref | 1.08 (0.86, 1.34), p=0.513 | 0.91 (0.69, 1.20), p=0.506 | 1.51 (1.17, 1.94), p=0.001 | 0.020 |
| Model 3^c^ | ref | 1.12 (0.87, 1.44), p=0.380 | 0.93 (0.68, 1.29), p=0.684 | 1.49 (1.10, 2.00), p=0.001 | 0.053 |

Data is presented with mean±SD or number of participants (percentage).

P<0.05 indicate significant difference between or across groups.

a Crude model.

b Adjusted for age, sex, and race/ ethnicity.

c Further adjusted for BMI, systolic blood pressure on right arm, smoking status, drinking status, TCHO, HDL-c and HbA1c.

Abbreviations: CVD, cardiovascular disease; NHANES, National Health and Nutrition Examination Survey; PAD, peripheral artery disease; BMI, body mass index; SBP, systolic blood pressure; TCHO, total cholesterol; HDL-c, high density lipoprotein cholesterol; HbA1c, glycated hemoglobin.

**Supplementary table 2.** Sensitivity analyses of the associations (hazard ratios, 95% confidence intervals) between ILSBPD and the risk of all-cause and cardiovascular mortality in general population (NHANES 1999-2004) using the first measured blood pressure and further adjusted for ABI.

| Model | Hazard ratio (95% CI), p value | | | | | |
| --- | --- | --- | --- | --- | --- | --- |
|  | Inter-leg systolic blood pressure, mmHg | | | | | |
|  | Continuous (per 5mmHg) | Groups |  |  |  |  |
|  |  | Group 1  (<5) | Group 2  (5-<10) | Group 3  (10-<15) | Group 4  (≥15) | P for trend |
| **All-cause mortality** | | | | | | |
| Deaths, No. /total, No. (%) | 2502/6590 (38.0%) | 861/2645 (32.6%) | 702/1912 (36.7%) | 369/980 (37.7%) | 570/1053 (54.1%) |  |
| Crude model ^a^ | 1.03 (1.03,1.03), p<0.001 | ref | 1.17 (1.06,1.29), p=0.002 | 1.19 (1.05,1.34), p=0.005 | 2.05 (1.84,2.27), p<0.001 | <0.001 |
| Adjusted model ^b^ | 1.00 (1.00,1.01), p=0.363 | ref | 1.05 (0.93,1.17), p=0.454 | 0.95 (0.82,1.10), p=0.478 | 1.11 (0.97,1.28), p=0.123 | 0.322 |
| **Cardiovascular mortality** | | | | | | |
| Deaths, No. /total, No. (%) | 639/6590 (9.7%) | 197/2645 (7.4%) | 172/1912 (9.0%) | 87/980 (8.9%) | 183/1053 (17.4%) |  |
| Crude model ^a^ | 1.04 (1.03,1.04), p<0.001 | ref | 1.25 (1.02,1.54), p=0.031 | 1.23 (0.95, 1.58), p=0.114 | 2.87 (2.35, 3.51), p<0.001 | <0.001 |
| Adjusted model ^b^ | 1.00 (0.99,1.01), p=0.68 | ref | 1.15 (0.90, 1.46), p=0.255 | 0.97 (0.72, 1.30), p=0.831 | 1.34 (1.02, 1.75), p=0.034 | 0.095 |

Data is presented with mean±SD or number of participants (percentage).

P<0.05 indicate significant difference between or across groups.

a Unadjusted model.

b Adjusted for BMI, systolic blood pressure on right arm, smoking status, drinking status, TCHO, HDL-c, HbA1c and ABI.

Abbreviations: ILSBPD, inter-leg systolic blood pressure difference; NHANES, National Health and Nutrition Examination Survey; ABI, ankle-brachial index; BMI, body mass index; SBP, systolic blood pressure; TCHO, total cholesterol; HDL-c, high density lipoprotein cholesterol; HbA1c, glycated hemoglobin.
